# Effectiveness of L-serine supplementation in children with a *GRIN2B* loss-of-function mutation: Rationale and protocol for single patient (n-of-1) multiple cross-over trials

**DOI:** 10.1016/j.conctc.2023.101233

**Published:** 2023-11-17

**Authors:** Bibiche den Hollander, Marieke Rothuizen-Lindenschot, Lisa Geertjens, Frédéric M. Vaz, Marion M. Brands, Hoang Lan Le, Agnies M. van Eeghen, Peter M. van de Ven, Martina C. Cornel, Bart A.W. Jacobs, Hilgo Bruining, Clara D. van Karnebeek

**Affiliations:** a Amsterdam UMC Location University of Amsterdam, Department of Pediatrics, Emma Children's Hospital, Amsterdam Gastroenterology Endocrinology Metabolism, Meibergdreef 9, Amsterdam, the Netherlands; b Emma Personalized Medicine Center, Amsterdam UMC, Amsterdam, the Netherlands; c United for Metabolic Diseases, Amsterdam, the Netherlands; d HAN University of Applied Sciences, Nijmegen, the Netherlands; e Amsterdam UMC Location Vrije Universiteit Amsterdam, Child and Adolescent Psychiatry and Psychosocial Care, Emma Children's Hospital, Boelelaan 1117, Amsterdam, the Netherlands; f Amsterdam UMC Location University of Amsterdam, N=You Neurodevelopmental Precision Center, Amsterdam Neuroscience, Amsterdam Reproduction and Development, Meibergdreef 9, Amsterdam, the Netherlands; g Amsterdam UMC Location University of Amsterdam, Department of Clinical Chemistry and Pediatrics, Laboratory Genetic Metabolic Diseases, Emma Children's Hospital, Meibergdreef 9, Amsterdam, the Netherlands; h Amsterdam Gastroenterology Endocrinology Metabolism, Inborn Errors of Metabolism, Amsterdam, the Netherlands; i Amsterdam UMC Location University of Amsterdam, Department of Core Facility Metabolomics, Meibergdreef 9, Amsterdam, the Netherlands; j Amsterdam UMC Location University of Amsterdam, Department of Hospital Pharmacy, Meibergdreef 9, Amsterdam, the Netherlands; k Medicine for Society, Platform at Amsterdam UMC, University of Amsterdam, 1105 AZ Amsterdam, the Netherlands; l Advisium, ‘s Heerlen Loo Zorggroep, Amersfoort, the Netherlands; m University Medical Center Utrecht, Department of Data Science and Biostatistics, Julius Center for Health Sciences and Primary Care, Heidelberglaan 100, Utrecht, the Netherlands; n Amsterdam UMC Location Vrije Universiteit van Amsterdam, Department of Human Genetics, Amsterdam Reproduction and Development, Meibergdreef 9, Amsterdam, the Netherlands; o Antoni van Leeuwenhoek, Department of Pharmacy and Clinical Pharmacology, Plesmanlaan 121, Amsterdam, the Netherlands; p Amsterdam UMC location University of Amsterdam, Department of Pediatrics, Emma Children’s Hospital, Amsterdam Public Health Research Institute, Methodology and Mental Health and Personalized Medicine, Meibergdreef 9, Amsterdam, the Netherlands; q Amsterdam UMC location University of Amsterdam, Department of Pediatrics, Emma Children’s Hospital, Amsterdam Reproduction & Development, Child Development, Meibergdreef 9, Amsterdam, the Netherlands

**Keywords:** GRIN2B_1_, L-serine_2_, Intellectual developmental disability_3_, N-methyl-D-aspartate receptor_4_, Personalized medicine_5_, n-of-1_6_

## Abstract

**Rationale:**

Loss-of-function (LoF) mutations in *GRIN2B* result in neurologic abnormalities due to *N*-methyl-D-aspartate receptor (NMDAR) dysfunction. Affected persons present with various symptoms, including intellectual developmental disability (IDD), hypotonia, communication deficits, motor impairment, complex behavior, seizures, sleep disorders and gastrointestinal disturbance. Recently, *in vitro* experiments showed that D-serine mitigates function to GluN2B (mutation)-containing NMDARs. 11 previous case reports are published on (experimental) L-serine treatment of patients between 1.5 and 12 years old with GRIN2B missense or null mutations, some of whom showed notable improvement in motor and cognitive performance, communication, behavior and abnormalities on electro encephalography (EEG). Our objective is to further evaluate the effectiveness of L-serine for *GRIN2B*-related neurodevelopmental disorder (*GRIN2B*-NDD), using an n-of-1 trial design, increasing the level of evidence.

**Methods/design:**

These n-of-1 trials, consisting of 2 cycles of 6 months, will be performed to evaluate the effect of L-serine compared to placebo in 4 patients with a *GRIN2B* LoF mutation. The aggregation of multiple n-of-1 trials will provide an estimate of the average treatment effects.

The primary outcome is the Perceive-Recall-Plan-Perform of Task Analysis, assessing developmental skills. Secondary outcomes include Goal Attainment Scaling, seizure log books, EEGs, sleep log books, the irritability subscale of the Aberrant Behavior Checklist, the Bristol Stool Scale and the Pediatric Quality of Life Inventory.

**Conclusion:**

This study employs an innovative methodological approach to evaluate the effectiveness of L-serine for patients with a GRIN2B LoF mutation. The results will establish a foundation for implementing L-serine as a disease-modifying treatment in GRIN2B-NDD.

## Highlights

• Following on case reports, we aim to assess L-serine's effectiveness in *GRIN2B*-NDD.
• N-of-1 trials serve as a type of RCT in individual patients.
• The synthesis of n-of-1 trial data can estimate average treatment effect.
• This trial will use innovative developmental outcome measures (PRPP-Assessment, GAS).
• Results inform larger L-serine trials as disease-modifying intervention for *GRIN2B*-NDD.

## Abbreviations:

ABC: Aberrant Behavior Checklist
EEG: electro encephalography
EMA: European Medicines Agency
FDA: Food and Drugs Administration
GAS: Goal Attainment Scaling
GRIN-NDD: GRIN2B-related neurodevelopmental disorder
GRD(s): GRIN-related disorder(s)
IDD: intellectual developmental disability
iGluR(s): ionotropic glutamate receptor(s)
IMD(s): inherited metabolic disorder(s)
LoF: loss-of-function
MB-CDIs: MacArthur-Bates Communicative Development Inventories
mGluR(s): metabotropic glutamate receptor(s)
NDD(s): neurodevelopmental disorder(s)
NMDAR(s): N-methyl-D-aspartate receptor(s)
PedsQL: Pediatric Quality of Life Inventory
PRPP-Assessment: Perceive-Recall-Plan-Perform System of Task Analysis
RCT(s): randomized controlled trial(s)

## 1 Introduction

### 1.1 N-of-1 trials

Randomized controlled trials (RCTs) in neurodevelopmental disorders (NDDs) pose specific challenges due to heterogeneity and rarity of conditions [1]. It is therefore difficult or even not possible to conduct adequately powered traditional RCTs to determine effectiveness. The n-of-1 methodology provides a promising alternative type of RCT. Controlled cross-over trials are executed within individual patients. At a trial's end for the individual, the randomization order is revealed and the treatment effect for the specific individual is estimated by comparing outcomes while on treatment versus control. By randomizing and varying treatment conditions within an individual patient, these trails generate robust evidence regarding the response to treatment in specific individuals [2,3]. This is especially valuable in cases where there is substantial heterogeneity and variability within the study population, such as in *GRIN2B*-related neurodevelopmental disorder (*GRIN2B*-NDD).

Aggregating the results of several n-of-1 trials provides an average treatment effect in the trial population [4,5]. This meta-analysis of individual trial data allows for a comprehensive assessment of the overall efficacy and generalizability of the intervention, despite the small sample sizes typically encountered in n-of-1 trials. This combined with the possibility to elucidate unique treatment response patterns and individual variations through analysis of individual-level data, facilitates the movement towards personalized care and proves a much-needed bridge between practice and science. This lead to robust evidence for treatment decisions at an individual level [3] and enhances future treatment predictions [6].

### 1.2 Definition of *GRIN2B*-NDD

*GRIN*-related disorders (GRDs) are a form of rare genetic encephalopathies and a novel group of inherited metabolic disorders (IMDs) [7]. GRDs are related to variations in ionotropic glutamate receptors and result from the presence of pathogenic variants in four of the seven *GRIN* genes (including *GRIN1* (OMIM#138249), *GRIN2A* (OMIM#138253), *GRIN2B* (OMaIM#138252), *GRIN2D* (OMIM#602717)), encoding for the *N*-methyl-D-aspartate receptor (NMDAR) subunits. Often variants in GRDs are *de novo* mutations and result in NMDAR loss-of-function (LoF). GRDs may present with varying debilitating consequences, as described below. Discrete *de novo* mutation of *GRIN2B* have been associated with NDD [[8], [9], [10]] such as early infantile epileptic encephalopathy/West syndrome [11].

### 1.3 Epidemiology

To date, about 400 individuals harbouring likely pathogenic *GRIN* variants have been reported worldwide (www.grin-database.de). The proportion of GRIN variants that represent LoF mutations is currently unknown. Around 120 of these 400 individuals entail mutations in *GRIN2B*. However, diagnostic panel sequencing in 2136 independent epilepsy patients revealed 7 (likely) pathogenic *GRIN2B* variants and a diagnostic frequency of 0.22 %. Similarly, 15 pathogenic/likely pathogenic *GRIN2B* variants were identified in 8051 patients with NDD, equaling similar frequency of 0.19 % [10]. Therefore, the GRD prevalence is probably underestimated and patients underdiagnosed.

### 1.4 Pathophysiology

Glutamate is the main excitatory amino acid neurotransmitter in the brain, with about 90 % of excitatory synapses using glutamate for neuronal communication [7]. Glutamate acts on a group of transmembrane receptors functionally separated into metabotropic (mGluRs) and ionotropic glutamate receptor (iGluRs) families. NMDARs are critical players of glutamatergic neurotransmission. Functionally, NMDARs play critical roles in neuronal survival, neurogenesis, synaptogenesis, and synaptic plasticity processes [12]. Concomitantly, disturbance of the NMDAR function, by means of genetic alterations, can affect glutamatergic neurons, circuits, brain activity, and with that neurocognitive functioning.

**Fig. 1:**
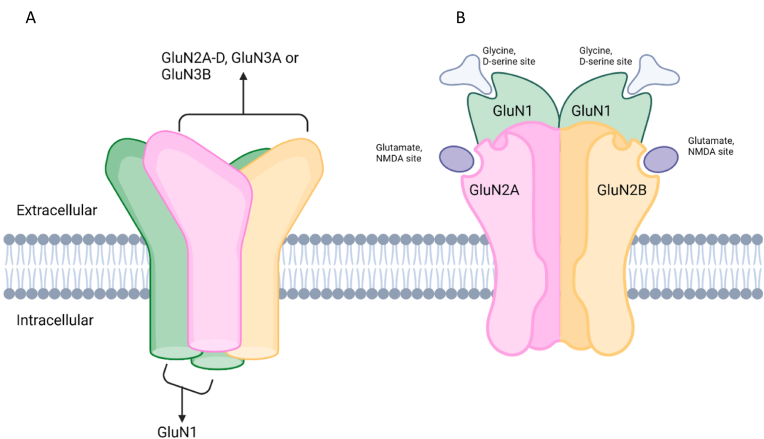
A: Schematic representation of the hetero tetrameric NMDA receptor: the receptor consists of four subunits. 1.B: The NMDA receptor is assembled as a tetramer and comprises of 2 GluN1 subunits along with 2 GluN2 or GluN3 subunits, of which there are 4 GluN2 subtypes (GluN2A, GluN2B, GluN2C, GluN2D) and 2 GluN3 subtypes (GluN3A, GluN3B). Glycine or D-serine binds to GluN1 (or GluN3) subunits, glutamate binds to GluN2 subunits. The activation of the NMDA receptor requires the presence of glutamate and also of a co-agonist: glycine or D-serine.

The NMDAR results from the oligomerization of two GluN1 subunits and a combination of two additional subunits (GluN2A-D, GluN3A, GluN3B) (Fig. 1) [13]. The subunit composition of NMDAR is spatially and temporally regulated with a switch from predominant GLUN2B expression in early development to more prominent synaptically localized GluN2A expression at later stages, which might explain the tendency toward earlier onset epilepsy phenotypes in *GRIN2B* mutation carriers [11]. It has been proposed that *GRIN2B* disturbance might markedly compromise critical steps of neuronal, synaptic, and brain circuitry development.

### 1.5 Clinical presentation

The neurodevelopmental condition in GRDs is manifested by a spectrum of neurological alterations, including IDD, hypotonia, communication impairment, movement disorders, complex behavior, seizures, sleep disorders, and gastrointestinal disturbances [10,11,14,15]. Specifically, *GRIN2B*-NDD is characterized by mild to profound developmental delay in all affected individuals. Muscle tone abnormalities (spasticity and/or hypotonia, occasionally associated with feeding difficulties), as well as epilepsy, autism spectrum disorder, and complex behavior are common. Other infantile- or childhood-onset findings include microcephaly, dystonic, dyskinetic, or choreiform movement disorder, and cortical visual impairment [16]. At present, the cohort size of patients with *GRIN2B*-NDD is too limited to establish robust genotype-phenotype correlations, which may be important for treatment stratification and prediction of therapeutic response.

### 1.6 Therapeutic interventions

*GRIN2B*-NDD is considered potentially treatable with variable clinical effect [17,18]. L-serine is the endogenous precursor of D-serine, and an endogenous NMDAR co-agonist [19]. L-serine is a naturally-occurring dietary amino acid. It is abundant in soy products, some edible seaweeds, sweet potatoes, eggs, and meat. L-serine is directly involved in the biosynthesis of purines, pyrimidines, and other amino acids. It has been approved as a normal food additive by the Food and Drug Administration (FDA) [20]. Further, L-serine supplement has been used in pediatrics at doses of 400–600 mg/kg/day for several decades for the treatment of a group of IMDs: 3-phosphoglycerate dehydrogenase deficiency, with no reported adverse effects [21].

The 2019 study by Soto et al. found that a *GRIN2B* LoF variant can be rescued *in vitro*, by means of elevated doses of D-serine administration [18]. More interestingly, they showed that supplementation of L-serine was associated with a significant improvement of motor and cognitive performance and communication in a pediatric patient with *GRIN2B*-related encephalopathy. A recent study in ten individuals with *GRIN2A*- or *GRIN2B*-related disorders who were treated with L-serine, reported improvements in behaviour in eight, in development in four, and/or in electro-encephalography (EEG) or seizures frequency in four [17]. In our previous study, we investigated the effect of L-serine in two patients, using an innovative functional outcome measure, the Perceive-Recall-Plan-Perform System of Task Analysis (PRPP-Assessment) [22]. This study showed an improvement in psychomotor development and cognitive function in one patient. In the most severely clinically affected patient no significant objective improvement in validated outcomes was observed.

Although initial studies suggest a positive effect of L-serine in some *GRIN2B*-NDD patients, more insights and evidence are needed. The rationale for selecting L-serine over D-serine in our study is firmly rooted in the existing literature and our interpretation of available evidence. As far as our current knowledge extends, the therapeutic approach in GRIN2B cases has involved the use of L-serine. It is noteworthy that the utilization of D-serine in the aforementioned study was exclusively for *in vitro* treatment [18].

Given the medical need for new therapies for the devastating neurological problems in these patients, further investigation of L-serine effectiveness in *GRIN2B*-NDD and other GRDs is warranted. Prospective placebo-controlled studies with personalized and sensitive outcomes are necessary given the clinical and genetic heterogeneity of the patient population. In addition, such a personalized methodological approach has the potential of maximizing treatment adherence [23,24]. Here we present the first double-blinded placebo-controlled n-of-1 trial protocol for L-serine in *GRIN2B*-NDD.

## 2 Methods and design

### 2.1 Study aims

This study will test the effectiveness of L-serine supplementation for improvement of developmental/functional outcomes, seizures, sleep, irritability, language, bowel movement and quality of life in children (≥37weeks gestational age, ≤18 years) with a *GRIN2B* LoF mutation compared to placebo by providing both individual and average treatment effect estimates.

### 2.2 Study design

**Fig. 2:**
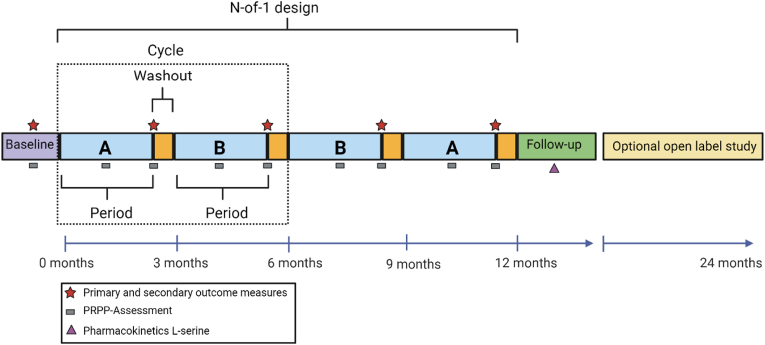
*Study design of the n-of-1 trial consisting of 2 cycles with randomization, a baseline period and an optional open label period*. *The follow-up consists of 2 appointments, at 6 weeks and* 3 months*.* A: L-serine, B: placebo.

A series of randomized, double blind, placebo-controlled, single patient (n-of-1) trials will be conducted in the Emma Center for Personalized Medicine at the Amsterdam UMC. Each participant will undergo two cycles, each of two treatment periods. The duration of each treatment period will be three months (+1 week washout), making a total of 13 months per participant to complete the full trial. The washout week will be added to the treatment period, and will not be used to assess effectiveness (half-life of L-serine is 17 h) [25]. In each cycle, L-serine or placebo will be randomly allocated to one of two treatment periods (Fig. 2). The decision to limit the study to two cycles is primarily predicated upon the anticipated timeframe necessary to discern substantial effects on our designated outcome parameters. Previous case series conducted by our research team have proved evidence that significant alterations in the measured outcomes are reasonably expected to manifest within a three-month duration [22]. Through careful calculation, we have determined that the inclusion of two cycles, each comprising two three-month intervals, totaling 12 months, allows us to capture the potential efficacy of the medication while optimizing the study's duration. This approach enables us to strike a delicate equilibrium between evaluating treatment effectiveness and curtailing the trial's length, a consideration of paramount importance for patient convenience and adherence. Furthermore, we remain attentive to the necessity of avoiding undue prolongation of the trial, thereby mitigating the burden on participating patients.

Outcome measures will be assessed during the baseline period and every 3 months during the whole trial period (the PRPP-Assessment every 1.5 month). Follow-up visits will be scheduled 6 weeks and 3 months after the end of the trial or after the end of the extension phase (see further). At the end of the trial, the order of medications is revealed, and outcome measures are compared between drug and placebo periods. The different n-of-1 trials will be aggregated to obtain an average estimate of the effect of L-serine.

After the two-cycle n-of-1 period, there is the option for the patient to continue into an open label period of one year. This gives patients the possibility to use L-serine for one extra year. Moreover, this enables us to analyze the results of the n-of-1 trial without withholding possible medication benefit. It will also provide more long-term information on the effects of L-serine. Outcome measures will be assessed in the open-label extension period after 6 and 12 months.

### 2.3 Study population and recruitment

Pediatric patients with a confirmed *GRIN2B*-NDD, referred to the Emma Center of Personalized Medicine at the Amsterdam UMC, will be considered potentially eligible. A maximum of four patients will be included due to cost restrictions. Patients will be invited to participate if they are aged between 0 (born ≥37 weeks gestational age) and 18 years old, and have a confirmed *GRIN2B* LoF mutation. The patient or the parent(s)/legal guardian(s) will provide written informed consent. Patients are excluded if they meet any of the following criteria: ingestion of L-serine within 30 days prior to enrolment; treatment with another investigational product within 30 days prior to enrolment; known hypersensitivity reactions, intolerance or adverse reactions to L-serine or the inactive ingredients; pregnancy; currently lactating; patient unwilling or, in the investigator's opinion, unable to adhere to the requirements of the study; unable to swallow powder and has no other enteral access; any condition or abnormality which may, in the opinion of the investigator compromise the safety of patients. The genetic variants of the four included patients are described in Table 1. Both the pathogenicity and variant type are derived from the results of the genetic research. The variant from patient 3 was also available in ClinVar. In our prior study, we delineated the methodology employed for conducting functional analyses of the variants and elucidating the loss-of-function [22].

**Table 1:** Overview of genomic data of the included four patients with GRIN2B-related NDD.

Genotypic spectrum	Patient 1	Patient 2	Patient 3	Patient 4
*GRIN* DNA variants	c.1583C > T	c.2087G > T	c.2459G > C^a^	c.2496C > G
Variant type (protein effect)	Missense	Missense	Missense	Missense
Amino acid change	p.(Pro528Leu)	p.(Arg820Leu)	p.(Gly820Ala)	p.(Ser832Arg)
Pathogenicity	Likely pathogenic	Likely pathogenic	Pathogenic/Likely pathogenic	Likely pathogenic
Functional analysis	Loss-of-function	Loss-of-function	Loss-of-function	Loss-of-function

a Genetic variant also present in ClinVar; ClinVar accessed in September 2023.

### 2.4 Primary outcomes

**Fig. 3:**
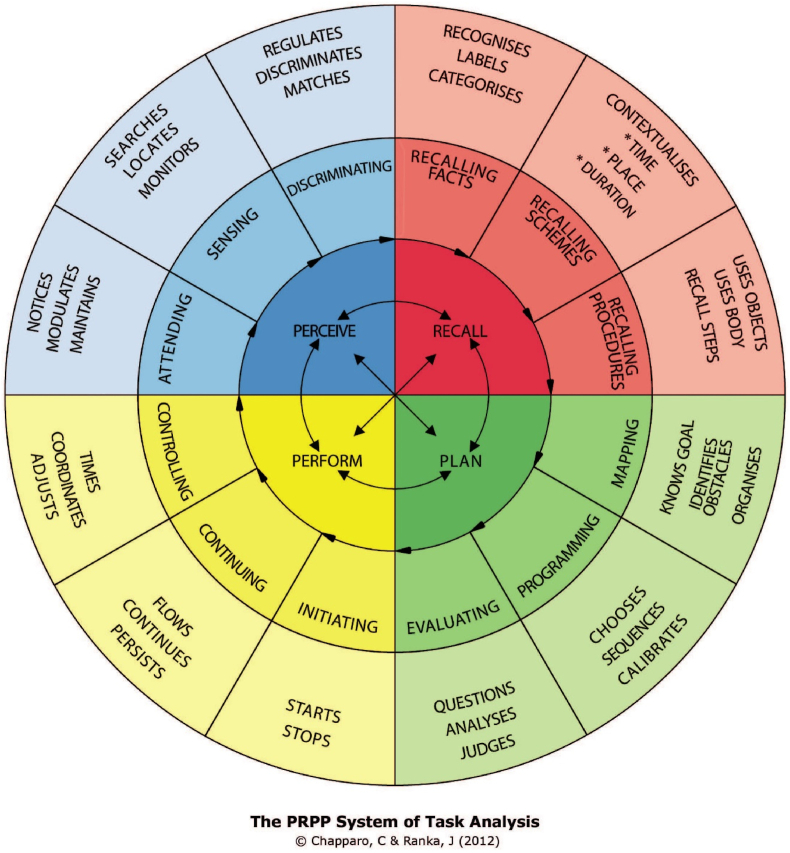
The perceive, recall, plan and perform (RPPP) system of task analysis conceptual model (Chapparo, C & Ranka, J 2012).

The PRPP-Assessment will be used to measure change in functional outcome (task mastery and cognitive strategy use). The PRPP-Assessment is a standardized, client-centered, performance-based, and criterion-referenced valid tool to assess the application of information processing strategies of a patient while performing meaningful everyday activities in a real-life environment [26,27]. This assessment is used by occupational therapists to gain insight into occupational performance by observing children performing meaningful activities in their everyday lives. The PRPP-Assessment is flexible, which makes it applicable for very different target-groups (i.e. different ages, different diagnosis, different levels of functioning etc.); the activity can be chosen by the child and/or parents, the activity is performed in the usual way in the actual context, and the assessment can be used with all different age groups and levels of functioning. The PRPP-Assessment uses a procedural task analysis (phase 1) and a process task analysis (phase 2), and can be performed in-person or via observation of videos. In phase 1, a procedural task analysis is used to divide the activity in relevant steps to assess if, and which type of, errors were made. As a result, phase 1 calculates an overall mastery score for the meaningful everyday activity. A percentage of 85 % mastery score on phase 1 is seen as an indicator for positive long-term sustained performance of the activity. Phase 2 of the PRPP-Assessment uses a cognitive task analysis that focuses on information processing strategies that are relevant for the execution of the task. It incorporates 35 items divided into the subscales of the PRPP-Assessment, which connects to a specific conceptual stage of information processing (Fig. 3). The 35 strategies are divided into attention and sensory perception (perceive quadrant) memory (recall quadrant), response planning and evaluation (plan quadrant), and performance monitoring (perform quadrant). Each cognitive strategy is evaluated on a three-point scale indicating how effectively the child used that cognitive strategy; resulting in a total score between 35 and 105 (higher score indicate more effective cognitive strategy application). In this study, the Dutch version of the PRPP-Assessment will be used, with scoring criteria according to the PRPP-Assessment manual that is instructed in the six-day PRPP assessment course for occupational therapists [28]. Per child, 2 activities will be measured (according to the guidelines). The videos are made by the parents/caregivers using the instructions from previous research [29]. Prior to filming, the activities are selected and the criterion is determined.

### 2.5 Secondary outcomes

The first secondary outcome is the Goal Attainment Scaling (GAS). GAS is used to identify and measure individualized goals related to the symptoms that are causing the greatest hindrance or difficulty for both parents and children [30]. GAS enables to focus on personalized and for participants relevant targets. GAS is an individualized outcome measure involving goal selection and goal scaling that is standardized in order to calculate the extent to which a patient's goals are met. Patients (if they are capable of doing so) and/or their caretakers are allowed to choose their own specific goals, coordinated and guided by their treating physician. This makes GAS a measurement instrument that has been proven to be sensitive to change, particularly in small heterogeneous groups [30].

Other secondary outcome measures include a seizure log book and resting state EEG. A baseline EEG will be performed, followed by 3-monthly measurements. The measurements will be used to detect changes in brain resting state EEG. EEG data will be pre-processed using Matlab software (MATLAB. (2022). *Version R2022a.* Natick, Massachusetts: The Mathworks Inc.) and processed offline using the Neurophysiological Biomarker Toolbox (http://www.ntbwiki.net/). With this toolbox, a wide array of resting state parameters can be evaluated including power spectra and coherence. In addition, we evaluate excitation-inhibition (E/I) ratios using a functional E/I measure. The basis of the E/I method is the finding that long-range temporal correlations weaken when network E/I is out of balance (based on computational and pharmacological testing), whereby the amplitude of oscillations tells us something about the direction of the disbalance [31]. This E/I method is validated at rest and after GABAergic treatment. In addition to a whole brain E/I measure, we aim to introduce source localization to gain better insight into the distribution of excitation and inhibition in the brain.

Additionally, secondary outcome measures include a sleep log book (hours of sleep, how often awake during the night), the Aberrant Behavior Checklist (ABC) irritability subscale [[32], [33], [34]], the MacArthur-Bates Communicative Development Inventories (MB-CDIs) [35], the Bristol stool scale, and the Pediatric Quality of Life Inventory (PedsQL) [36]. Other data include adverse events, safety parameters in plasma, vital signs and physical examination and pharmacokinetics of L-serine. Finally, plasma concentrations of L-serine and D-serine in all patients will be assessed in the Laboratory Genetic Metabolic Diseases of the Amsterdam UMC. Based on two previous studies, blood samples for pharmacokinetics analyses will be collected at 0 (pre-dose), 60, and 150 min after L-serine ingestion during the extension phase [37,38].

### 2.6 Randomization

The trial is composed of two cycles, each with two treatment periods of three months and one week washout, giving a total duration of 13 months. The random allocation sequence will be generated using block randomization in a 1:1 ratio with blocks of size two and implemented by the unblinded hospital pharmacist. Participants and investigators will be blinded for allocation.

### 2.7 Safety reviews

At each contact with the researcher, adverse events will be recorded. Parents and patients will be instructed to contact the research team in the case of any adverse events occurring.

Seriousness, causality, severity and expectedness of adverse events will be evaluated. Cases that are considered serious, possibly, probably or definitely related to the drug and are unexpected are to be unblinded. Any study-related problem posing risk of harm to participants and any type of serious adverse event will be reported to the institutional ethics committee. Despite L-serine being a dietary supplement, it is considered a drug in this context. The regulations applicable to drug studies are therefore applicable to this study as well.

### 2.8 Treatment, concomitant medications and compliance

The study drug to be tested is oral L-serine versus placebo. L-serine capsules (or placebo capsules) are administered orally with a fluid or enterally by naso-/gastro-intestinal tube between meals. Based on the previous study of Soto et al., it will be administered at a target dose of 500 mg/kg/day, divided in four equal doses [18]. The pharmacist of the Amsterdam UMC will produce and package pharmaceutical grade L-serine and matching placebo capsules in compliance with Good Manufacturing Practice for Investigational Medicinal Products. Drug accountability will be determined by a medication diary. There are no known medications or foods that interfere with the metabolism of L-serine.

### 2.9 Post-trial treatment for individual patients

Once the analysis has been completed for a specific child, a report detailing the findings, adverse events during placebo and treatment, and recommendations to continue or stop treatment with the trial medication will be written. An appointment is made for the child and its parents or caretakers to discuss the results and make a decision regarding further treatment with L-serine for one extra year.

### 2.10 Statistical considerations

#### 2.10.1 Sample size

The sample size of four patients is based on clinical considerations and has no formal statistical basis. The PRPP-Assessment showed considerable improvement in one previous patient with a LoF *GRIN2B* mutation treated with L-serine (PRPP mastery score of 10 % at baseline, PRPP mastery score of 78 % after 12 months) [22]. Therefore, we expect that we might be able to demonstrate an effect of L-serine in this series of four patients provided that the effects are indeed very large.

#### 2.10.2 Statistical analysis

Baseline characteristics of the included subjects will be described and we will produce a flow diagram describing the flow of participants through the trial processes. In general, because of the small sample size, descriptive statistics will be used to summarize the individual and average treatment effects for the primary and secondary outcomes. The primary outcome comparison and estimate for the average treatment effect will be based on the difference in the mean PRPP-Assessment at the end of each treatment period averaged over the two cycles (separately for phase 1 and 2 of the assessment). The analysis will be based on a linear mixed model that will contain a fixed effect for treatment (L-serine/placebo), and random intercept and random treatment effect for patient and a random effect for cycle within patient. Analyses will be performed in R, using the Imer package. In case the mixed model analysis fails to converge, the summary measures analysis approach will be used [39]. If needed to satisfy the assumptions of normality, a suitable transformation will be applied. A two-sided significance level of 5 % will be used and 95 % confidence intervals for the average treatment effect will be provided.

A mixed model analysis with similar model structure will be performed for the average treatment effects on the secondary outcomes.

L-serine and D-serine plasma concentrations will be summarized using descriptive statistics. Adverse events, abnormalities in safety parameters, vitals, height, weight, and other abnormalities found in physical examination will be reported.

## 3 Ethical considerations

### 3.1 Ethical approval

Written approval has been obtained from the Amsterdam UMC Ethics Committee prior to study commencement (30 June 2022, 2022.0271 – NL80290.018.22). Written informed consent is required from the parent or caretakers (and assent for children aged >12 years and capable of making informed decisions). All patients receive a re-identifiable registration number for the trial CRFs and database.

### 3.2 Trial withdrawal and discontinuation of trial medication

Patients can withdraw from the trial at any time without mentioning a reason and without impact on usual care. Provided reasons for withdrawal will be recorded. Only data from completed cycles will be included in the analysis.

## 4 Discussion

GRDs belong to an expanding and heterogeneous group of IMDs, with inherent challenges in effective treatment. A personalized approach with improved therapeutic interventions is urgently needed to tackle these challenges and improve patient outcomes [40]. Recruiting sufficient numbers of children who have a *GRIN2B* LoF mutation in traditional RCTs is problematic, and averaging effect size in such a heterogeneous disorder is often unreliable. Therefore, we propose an alternative methodology to generating high-level evidence by using aggregated n-of-1 trials to assess the effectiveness of L-serine on symptoms in *GRIN2B*- NDD. Previous studies on the effect of L-serine in patients with *GRIN2A-* and *GRIN2B-*related NDD are non-blinded, non-randomized case studies with no placebo [17,18,22]. Hence, this study will provide additional high-quality evidence.

Due to their cross-over design, and the fact that the patient is his/her own control, aggregated n-of-1 trials have a smaller required sample size than their conventional parallel arm RCT counterparts for equivalent levels of statistical power, and are better at controlling for confounding [41]. As every participant receives both the active and placebo treatment, this makes participation more attractive than in a conventional RCT where there is a chance of being randomized to the placebo arm of the trial. Finally, because the same person contributes multiple data points to both the active and placebo arms of the trial, the sample is perfectly matched.

An additional strength of this proposed study is that for each child participant, the individual response is measured, instead of the mean response, like in traditional RCTs. As a result, a personalized report on efficacy and adverse effects can be provided to the parents. Additionally, the primary outcome measure is at the activity level, aiming to capture not only improvements in function but also their meaningful impact on the patient's daily life.

This study will be the first to use aggregated n-of-1 trials in a pediatric population with *GRIN2B* LoF mutations. An important component of the study is not only to test the effectiveness of L-serine on the included outcomes in children, but to evaluate the methodology and analytical aspects of employing this methodology in patients with GRDs. This information will contribute to proof-of-concept and ensure the acceptance of this method as a valuable and reliable research tool especially in drug trials where populations are difficult to recruit. As no previous n-of-1 trials have been conducted in the area of GRDs, the proposed protocol is expected to considerably enhance GRD knowledge, therapy and overall practice and contribute to approval for reimbursement. The accumulated evidence in the literature supports the utility of n-of-1 trials evaluating treatment effectiveness in rare conditions in a patient-centered manner [42]. Furthermore, the European Medicines Agency (EMA) increasingly acknowledges the significance of personalized trials and has been placing greater emphasis on issuing directives to guide their conduct and interpretation [43]. The Oxford Centre for Evidence-Based Medicine has designated n-of-1 trials as Level 1 evidence, placing them on par with systematic reviews of RCTs [44]. Nevertheless, there are numerous essential strides yet to be taken in order to integrate n-of-1 trials into widespread practice and deliver substantial benefits to patients and the healthcare system [45].

## Funding

L-serine and placebo capsules were supported by platform Medicine for Society. This work was supported by United for Metabolic Disease [UMD-CG-2021-019, 2021], ‘s Heeren Loo. Stichting Metakids NL [2020-01-UMD, 2020], and Ladies Circle Nederland to CK and MB. BJ is member of the platform Medicine for Society, for which funding is provided by the Postcodeloterij. AvE is member of the European Reference Network ITHACA. LG and HG are supported by NWA.1160.18.200 NewTDech from NWO (Dutch Science Organisation).

## Ethics

The current study has been approved by the Medical Ethics Board of the Amsterdam UMC location University of Amsterdam (registration number: 2022.0271).

## Author contributions

Bibiche den Hollander: conceptualization, methodology, writing-original draft, writing-review & editing, project administration. Marieke Rothuizen-Lindenschot: methodology, writing-review & editing. Lisa Geertjens: methodology, writing-review & editing. Frédéric M. Vaz: methodology, writing-review & editing. Marion M. Brands: conceptualization, methodology, writing-original draft, writing-review & editing, supervision, project administration, funding acquisition. Hoang Lan Le: methodology, writing-review & editing. Agnies M. van Eeghen: methodology, writing-review & editing. Martina C. Cornel: methodology, writing-review & editing. Bart A.W. Jacobs: methodology, writing-review & editing. Hilgo Bruining: methodology, writing-review & editing, supervision. Clara D. van Karnebeek: conceptualization, methodology, writing-original draft, writing-review & editing, supervision, project administration, funding acquisition

## Data statement

Data sharing is not applicable to this article as no new data were created or analyzed in this study.

## Declaration of competing interest

The authors declare that they have no known competing financial interests or personal relationships that could have appeared to influence the work reported in this paper.
