# Corrigendum to “Effectiveness of L-serine supplementation in children with a GRIN2B loss-of-function mutation: Rationale and protocol for single patient (n-of-1) multiple cross-over trials” [Contemp. Clin. Trials Commun. 36 (2023)/ DOI: 10.1016/j.conctc.2023.101233]

**DOI:** 10.1016/j.conctc.2024.101264

**Published:** 2024-02-01

**Authors:** Bibiche den Hollander, Marieke Rothuizen-Lindenschot, Lisa Geertjens, Frédéric M. Vaz, Marion M. Brands, Hoang Lan Le, Agnies M. van Eeghen, Peter M. van de Ven, Martina C. Cornel, Bart A.W. Jacobs, Hilgo Bruining, Clara D. van Karnebeek

**Affiliations:** a Amsterdam UMC Location University of Amsterdam, Department of Pediatrics, Emma Children's Hospital, Amsterdam Gastroenterology Endocrinology Metabolism, Meibergdreef 9, Amsterdam, the Netherlands; b Emma Personalized Medicine Center, Amsterdam UMC, Amsterdam, the Netherlands; c United for Metabolic Diseases, Amsterdam, the Netherlands; d HAN University of Applied Sciences, Nijmegen, the Netherlands; e Amsterdam UMC Location Vrije Universiteit Amsterdam, Child and Adolescent Psychiatry and Psychosocial Care, Emma Children's Hospital, Boelelaan, 1117, Amsterdam, the Netherlands; f Amsterdam UMC Location University of Amsterdam, N=You Neurodevelopmental Precision Center, Amsterdam Neuroscience, Amsterdam Reproduction and Development, Meibergdreef 9, Amsterdam, the Netherlands; g Amsterdam UMC Location University of Amsterdam, Department of Clinical Chemistry and Pediatrics, Laboratory Genetic Metabolic Diseases, Emma Children's Hospital, Meibergdreef 9, Amsterdam, the Netherlands; h Amsterdam Gastroenterology Endocrinology Metabolism, Inborn Errors of Metabolism, Amsterdam, the Netherlands; i Amsterdam UMC Location University of Amsterdam, Department of Core Facility Metabolomics, Meibergdreef 9, Amsterdam, the Netherlands; j Amsterdam UMC Location University of Amsterdam, Department of Hospital Pharmacy, Meibergdreef 9, Amsterdam, the Netherlands; k Platform Medicine for Society, Amsterdam, the Netherlands; l Advisium, ‘s Heerlen Loo Zorggroep, Amersfoort, the Netherlands; m University Medical Center Utrecht, Department of Data Science and Biostatistics, Julius Center for Health Sciences and Primary Care, Heidelberglaan 100, Utrecht, the Netherlands; n Amsterdam UMC Location Vrije Universiteit van Amsterdam, Department of Human Genetics, Amsterdam Reproduction and Development, Meibergdreef 9, Amsterdam, the Netherlands; o Antoni van Leeuwenhoek, Department of Pharmacy and Clinical Pharmacology, Plesmanlaan 121, Amsterdam, the Netherlands

The authors regret the need for the following corrigendum:

In table 1, the amino acid change for the patient is listed incorrectly. It should be p.(Arg696Leu) instead of p.(Arg820Leu).

The authors would like to apologise for any inconvenience caused.

